# Maternal characteristics associated with injury-related infant death in West Virginia, 2010-2014

**DOI:** 10.1371/journal.pone.0220801

**Published:** 2019-08-12

**Authors:** Wilson A. Koech, Toni M. Rudisill, Ian R. H. Rockett

**Affiliations:** 1 Department of Epidemiology, School of Public Health, West Virginia University, Morgantown, West Virginia, United States of America; 2 Department of Psychiatry, University of Rochester Medical Center, Rochester, New York, United States of America; CUNY School of Medicine, City College of New York, UNITED STATES

## Abstract

Although injury-related deaths have been documented among children and adult populations, insufficient attention has been directed towards injury-related infant deaths. The objective of this retrospective study was to investigate maternal and infant characteristics associated with injury-related infant deaths in West Virginia. Birth and infant mortality data for 2010–2014 were sourced from the West Virginia Bureau for Public Health, Charleston. Relative risk was calculated using log-binomial regression utilizing generalized estimating equations. Maternal characteristics associated with injury-related infant mortality in West Virginia were race/ethnicity ($X_{\mathrm{df}=2}^{2}$ = 7.48, p = .03), and smoking during pregnancy ($X_{\mathrm{df}=1}^{2}=13.1$, p < .00). Risk of a Black Non-Hispanic infant suffering an injury-related death was 4.0 (95% CL 1.7, 9.3) times that of infants of other races/ethnicities. Risk of an infant dying from an injury-related cause, if the mother smoked during pregnancy, was 2.9 (95% CL 1.6, 5.0) times the risk of such a death if maternal smoking status during pregnancy is unknown or no smoking, controlling for race/ethnicity. This study provides important information to public health stakeholders at both the state and local levels in designing interventions for partial reduction or prevention of injury-related infant mortality in West Virginia.

## Introduction

Unintentional injury deaths and homicides impact children across all ages in the United States (U.S).[1–3] Although the unintentional injury-related death rate among children 19 years or less declined by 29% from 2000 to 2009 nationally, the corresponding unintentional injury-related infant mortality rate rose approximately 20%.[4] The smallest decline in unintentional injury-related infant mortality was observed among the Black population (-21%). Between 1999 and 2004, the national unintentional injury death rate increased by 7%,[5] and for the period 2000–2009 there was an annualized national rate of 1,081 infant deaths per 100,000 population.[6] Overall child mortality during this period in the U.S was documented to be 11,561 children deaths per 100,000 population, whereas the rate for West Virginia was 597 per 100,000.[6]

Overall, the rate of both unintentional and intentional injury-related deaths rose by 10% between 2000 and 2009 in the U.S. During the same period, children aged 0–4 years registered an injury-related death rate of 18.3 deaths per 100,000 population,[7] but by 2014, unintentional injury was the fifth leading cause of infant mortality in the U.S,[8, 9] and the fourth leading cause in West Virginia.[10]

Known risk factors associated with injury-related infant mortality include unhealthy behaviors associated with financial hardship, parental/caregiver behavior, and birth order.[11–14] A study based on California’s metropolitan areas showed an increase in the unemployment rate of 1% was associated with an increase of 8% in the unintentional injury-related infant mortality rate.[12] Parental behaviors, such as inappropriate child car-seat placement, are associated with increased risk of unintentional injury-related infant mortality in the U.S.[12] Similarly, caregiver behavior, such as non-adherence to infant injury prevention recommendations (e.g., not having infants sleep in the prone position or leaving an infant unattended on a raised surface), is associated with elevated risk.[13] Head injuries are frequent among children involved in motor vehicle accidents, irrespective of age.[11] During 2003–2013 period, unintentional injuries increased by 11%,[15] and Black infants were two to three times more likely to die from all injury, unintentional injury and homicide compared to White infants.[16] In the U.S., non-first born children have an increased risk of injury-related death.[14]

Even though high rates of injury-related infant mortality are being observed in the U.S and in West Virginia, current prevention efforts in West Virginia are inadequate. The focus of this project has been maternal and infant characteristics associated with injury-related infant death in West Virginia, adjusted for race/ethnicity. Understanding maternal and infant characteristics associated with injury-related infant deaths could inform public health stakeholders on potential interventions that could partially prevent such deaths in West Virginia, an extremely poor state and the only one in the U.S. that is fully immersed in Appalachia. The Appalachian region is characterized by poor access to healthcare, low income, and high rates of unemployment and poverty.[17]

## Materials and methods

### Data source

The primary data source for this analysis was birth and death records obtained from the West Virginia Bureau for Public Health, Charleston. The West Virginia University Institutional Review Board approved the parent study.

### Dependent variable

The project outcome was a binary variable labeled injury-related infant deaths (0 = alive, 1 = died). Injury-related infant deaths were constructed from external causes of mortality, building on the Centers for Disease Control and Prevention (CDC) injury classification matrix.[18] In this matrix, injury-related infant deaths by manner of injury are: 1) accidents by cutting/piercing, drowning, fall, fire/hot object or substance, firearm, machinery, and transport; 2) homicides by cutting/piercing, drowning, fall, fire/hot object or substance, firearm, and transport; 3) other (legal intervention/war, and undetermined). Therefore, the outcome encompasses infant deaths by manner of injury, categorized by ICD-10 revision codes V01-X59, Y85-Y86, X85-Y09, Y87.01, 87.02, Y87.1, Y10-Y34, Y87.2, Y89.9, Y35, Y36, Y89.0, and Y89.1. Excluded from the outcome were infant deaths associated with other causes.

### Independent variables

Explanatory variables included both categorical and continuous. These variables were: 1) maternal demographic, health, and behavior characteristics including education (greater than high school, high school/GED or less), insurance type/payer (Medicaid, Non-Medicaid and unknown), smoking during pregnancy (yes, no, unknown), age, race-ethnicity (Black non-Hispanic, other races/ethnicities), pregnancy term (preterm, term or post-term), trimester primary care began (first, second, third, no care), gestational diabetes (yes, no) and gestational hypertension (yes, no); and 2) newborn characteristics, including birth year, sex, and birthweight.

### Statistical analysis

The statistical analysis employed both descriptive and inferential statistics. Among descriptive statistics captured were variables proportions, mean, and spread. Furthermore, statistical investigation covered variable correlation analysis using Kendell correlation and multi-collinearity was assessed.

In inferential statistics, maternal and infant characteristics associated with injury-related deaths, as well as risk of injury-related mortality, were investigated. Relative risk was calculated using log-binomial regression utilizing generalized estimating equations (GEE).[19] Bivariable analysis was conducted, and main effects with p>0.15 were included in the model. Thereafter, a parsimonious model was developed and compared with an intercept-only model using Akaike’s Information Criterion (AIC), and two-way interaction effects for all variables were considered. In addition, model fitness was investigated using deviance. Likewise, Wald chi-square statistics and associated 95% confidence limits were reported.

Data management and analysis were conducted using SAS/STAT software, version 9.4 of the SAS systems for windows. Copyright 2002–2012 by SAS Institute Inc., Cary, NC, USA.[20]

## Results

The West Virginia live birth distribution for the period 2010–2014 by race/ethnicity was 93.6% White (non-Hispanic), 3.5% Black (non-Hispanic), and 2.9% other races/ethnicities. A majority of injury-related infant deaths were White. When infant deaths were stratified by form of payment during the perinatal period, 41% of women utilized the Medicaid insurance program. When stratified by maternal smoking during pregnancy, 49% of the infant deaths were to the women who smoked (Table 1).

**Table 1:** Maternal and infant characteristics of West Virginia live births, 2010–2014, n = 102,683.

Variable	Class	Died	Alive
		%*	%*
Race/ethnicity	White Non-Hispanic	86.0	93.6
	Black Non-Hispanic	12.0	3.5
	Other	2.0	2.9
Pregnancy term	Term & Post-term	88.0	89.3
	Preterm	12.0	10.7
Sex	Male	54.9	50.9
	Female	45.1	49.1
Prenatal Care	Prenatal	98.0	99.4
	No care	2.0	0.6
Insurance status	Non-Medicaid or Unknown	58.8	50.2
	Medicaid	41.2	49.8
Smoke during pregnancy	No or Unknown	51.0	73.6
	Smoke	49.0	26.4
Maternal Education	≤ High School	47.1	48.2
	> High School	52.9	51.8
Gestational Hypertension	No	92.2	93.0
	Yes	7.8	7.0
Gestational Diabetes	No	98.0	95.6
	Yes	2.0	4.4
Maternal age-group	≤ 20 years	19.6	17.4
	21–39 years	78.4	81.3
	≥ 40 years	2.0	1.3

* = Column percentage relative to variable class, totaling to 100% per variable.

Data source: West Virginia Bureau for Public Health, Charleston, WV

Investigation of explanatory variable correlation was implemented using the Kendell correlation, and multicollinearity was assessed using the variance inflation factor (data not shown). A bivariable binomial regression analysis was executed to identify significant main effects used in developing a fully parameterized model. Significant main effects in the bivariable regression analysis were race/ethnicity (p = .0311), smoking during pregnancy (.0006), and insurance status (p = .1198).

Significant main effects in the multivariable log-binomial regression model using GEE were race/ethnicity, $X_{\mathrm{df}=2}^{2}$ = 7.48, p = .0276, and smoking during pregnancy, $X_{\mathrm{df}=1}^{2}=13.1$, p = .0003. See Table 2 for more detail. Overdispersion was assessed using model deviance, and model fitness was assessed using AIC. The intercept only model AIC was 846.1, and the parsimonious model AIC was 26.6, meaning the parsimonious model provided better predictive power than the intercept-only model.[21]

**Table 2:** Relative risk for maternal characteristics associated with injury-related infant deaths, WV 2010–2014.

Parameter	Class	Relative Risk	Relative Risk 95% CL*		x^2^	p-value
			Lower	Upper		
Race/ethnicity	Black Non-Hispanic	4.0	1.7	9.3	10.1	0.0015
Smoking during pregnancy	Yes	2.9	1.6	5.0	13.7	0.0002

Race/ethnicity = (Black Non-Hispanic, Other races/ethnicities)

Smoking during pregnancy = (Yes or No, and unknown)

* = 95 percent confidence limits.

Data source: West Virginia Bureau for Public Health, Charleston, WV.

Explanatory variables assessed using log-linear regression model are race/ethnicity, smoking during pregnancy, birthweight, Pregnancy term, sex prenatal care, insurance status, maternal education, maternal age group, and all possible two-way interactions.

The risk of a Black infant dying from injury-related causes was 4.0 (95% CL 1.7, 9.3) times the risk for infants from other races/ethnicities, adjusting for other factors in the model. However, the adjusted risk of an infant dying from injury-related causes if the mother smoked during pregnancy was 2.9 (95% CL 1.6, 5.0) times the risk if smoking status was unknown or the mother did not smoke (Table 2).

## Discussion

This study evaluated maternal and infant characteristics associated with injury-related infant death in West Virginia. Race/ethnicity and smoking during pregnancy were significantly associated with injury-related infant deaths in West Virginia. Similar findings of racial/ethnic disparity in injury-related deaths are documented by Khan and colleagues, and by Ahrens and colleagues.[3, 14] Such differences in injury-related infant mortality might be explained by racial/ethnic disparities in healthcare access and utilization. Past studies showed that Black non-Hispanics were more likely than White Non-Hispanic counterparts to receive low-quality healthcare and to have higher risk of hospital death following injury.[16, 22] Racial/ethnic disparities in injury-related infant deaths highlight the need for tailored intervention strategies that will improve healthcare access at state and local levels.

Smoking during pregnancy was significantly associated with injury-related infant mortality. This variable might be a proxy for capturing characteristics known to be associated with injury-related deaths, such as infant maltreatment.[23] Cigarette smoking has been associated with drug abuse.[24] Therefore, smoking during pregnancy might be a proxy for substance abuse, a risk factor for child neglect and maltreatment.[25] It was expected to be statistically significant risk factor for infant mortality because child neglect and maltreatment is associated with drug abuse.[24, 25]

Insurance status in the perinatal period was an important maternal characteristic to investigate. Although insignificant in the multivariable analysis, it was significant in the bivariable analysis (p = 0.0566), and warrants further investigation using balanced racial/ethnic populations, since socio-economic disparities are associated with injury-related deaths by manner.[26]

Study limitations include the small number of cases of injury-related deaths, the highly skewed nature of the racial/ethnic distribution in West Virginia, and deletion of infant deaths associated with other causes. Small numbers can destabilize statistical models. Therefore, findings from this study should be interpreted with caution. Detailed presentation of descriptive statistics, such as injury-related infant mortality rates, could not be presented. CDC opposes publication of sample size, cell sizes or mortality rates involving fewer than ten cases.[27] The reliability of vital records might be also be problematic due to case misclassification. Past research documents sources of errors in medical records collected for purposes other than research.[28] Finally, variables collected in vital records are limited, yielding fewer options for statistical model building. In addition, no paternal characteristics were collected for the period of pregnancy, thus the study might be missing some important albeit unknown information.

## Conclusion

Black (non-Hispanic) infants in West Virginia have higher odds of experiencing injury-related mortality compared to infants of other races/ethnicities. Furthermore, infants of women reporting smoking during pregnancy have higher odds of injury-related death than those of women who reported not smoking or whose smoking status was unknown.

This study provides important information to public health stakeholders at both state and local levels. Study implications include formulating community-based intervention strategies to protect at-risk populations from lost years of life associated with injury-related infant mortality. Future research should investigate the association between all household characteristics and injury-related infant deaths. This study could only access maternal and infant characteristics, and paternal characteristics could provide additional important information.
